# Identifying Key Drivers of Peatland Fires Across Kalimantan's Ex‐Mega Rice Project Using Machine Learning

**DOI:** 10.1029/2021EA001873

**Published:** 2021-11-24

**Authors:** Alexander J. Horton, Vili Virkki, Anu Lounela, Jukka Miettinen, Sara Alibakhshi, Matti Kummu

**Affiliations:** ^1^ Department of Built Environment Aalto University Espoo Finland; ^2^ Development Studies, Social and Cultural Anthropology University of Helsinki Helsinki Finland; ^3^ VTT Technical Research Centre of Finland Ltd. Espoo Finland; ^4^ Department of Geosciences and Geography University of Helsinki Helsinki Finland

**Keywords:** Peatland forest fires, random forest XGBoost, anthropogenic impact, land cover, land use change

## Abstract

Throughout Indonesia ecological degradation, agricultural expansion, and the digging of drainage canals has compromised the integrity and functioning of peatland forests. Fragmented landscapes of scrubland, cultivation, degraded forest, and newly established plantations are then susceptible to extensive fires that recur each year. However, a comprehensive understanding of all the drivers of fire distribution and the conditions of initiation is still absent. Here we show the first analysis in the region that encompasses a wide range of driving factors within a single model that captures the inter‐annual variation, as well as the spatial distribution of peatland fires. We developed a fire susceptibility model using machine learning (XGBoost random forest) that characterizes the relationships between key predictor variables and the distribution of historic fire locations. We then determined the relative importance of each predictor variable in controlling the initiation and spread of fires. The model included land‐cover classifications, a forest clearance index, vegetation indices, drought indices, distances to infrastructure, topography, and peat depth, as well as the Oceanic Niño Index (ONI). The model performance consistently scores highly in both accuracy and precision across all years (>75% and >67.5% respectively), though recall metrics are much lower (>25%). Our results confirm the anthropogenic dependence of extreme fires in the region, with distance to settlements and distance to canals consistently weighted the most important driving factors within the model structure. Our results may help target the root causes of fire initiation and propagation to better construct regulation and rehabilitation efforts to mitigate future fires.

## Key Points

- The primary driving factors affecting fires in the ex‐Mega Rice Project area are anthropogenic
- The predictive capability of fire models is limited by the influence of human agency on the location of fire ignition
- More comprehensive understanding of the driving social factors affecting fire initiation are required

## 1 Introduction

In recent decades, anthropogenic ecosystem degradation, agricultural expansion, and the draining of peatland has transformed large areas of Indonesian tropical swamp forests into degraded peatlands, which are increasingly vulnerable to recurrent fires. Since the late 1990s, fires have become large‐scale occurrences that precipitate societal and livelihood changes (Lounela, 2021; Suyanto et al., 2009), release enormous quantities of stored carbon into the atmosphere (Huijnen et al., 2016; Page et al., 2002; van der Werf et al., 2017), spread haze pollution and health problems across a wide area (Marlier et al., 2015), and induce profound landscape alterations and ecosystem degradation (Miettinen, Hooijer, et al., 2012, Miettinen, Shi, et al., 2012).

The recurrent destruction of peatland by fires exacerbates local populations' vulnerabilities, raising tensions, and forcing a shift from subsistence livelihoods to new and often precarious practices in an effort to transform the degraded peatlands into an economically productive landscape (Goldstein, 2020; Lounela, 2021; Medrilzam et al., 2014). Tropical peatland fires also represent huge economic losses, with the estimated loss of income to the Indonesian economy due to the 2015 fires in terms of destruction of property, infrastructure, and potential earnings from lost crop yields and trade surpassing 16 billion USD (1.8% of Indonesia's GDP; World Bank, 2015), as well as damage to human health through smoke and haze pollution (Gaveau et al., 2015; Heil et al., 2006; Marlier et al., 2015). In addition to economic and social impacts, peatland fires have huge climate impacts, as they represent one of the largest reserves of near surface terrestrial organic carbon globally containing between 90 and 180 Gt of carbon (Friedlingstein et al., 2020; Grace et al., 2014; Hodgkins et al., 2018; Moore et al., 2013; Page et al., 2011). The persistent fires that are now an annual occurrence have the potential to convert these carbon stores into some of the largest carbon sources by releasing the terrestrial carbon locked in peatlands into the atmosphere during fires (Field et al., 2009; Hooijer et al., 2012). Both in 1997 and in 2015, exceptionally severe fires across tropical peatland regions in Southeast Asia released between 0.81–2.57 Gt of carbon into the atmosphere, equivalent of to up to 40% of total global fossil fuel emissions within those years (Huijnen et al., 2016; Lohberger et al., 2018).

The location for this study is the southern portion of the Central Kalimantan Province on the island of Borneo, which exhibits the highest density of peatland fires in Southeast Asia (Miettinen et al., 2017). The province of Central Kalimantan covers around 15 million hectares, of which 17% is peatland (2.5 million ha). In legal terms, the designated area for the state owned forest forms about 80 percent of the province, but only a small part of the area now has forest cover (as shown on the Indonesian Central Bureau of Statistics website retrieved 15.1.2021: https://www.bps.go.id/). Compared to other provinces in Indonesia, the deforestation rate in Central Kalimantan is high, with more than 200,000 hectares lost per year between 2013–2017 (Forest Watch Indonesia 2019). Since 1997 forest fires have been a recurring phenomenon due to decades of logging, oil palm plantation development, and an unsuccessful large‐scale rice cultivation scheme known as the Mega Rice Project that inadvertently transformed swamp forests into degraded peatlands.

In 2010, Central Kalimantan was nominated as a climate change pilot province by the central government and President Susilo Bambang Yudhoyono. This has prompted a number of climate mitigation strategies and peatland rehabilitation schemes in the area by international donors and local NGOs in collaboration with state agencies in an attempt to reduce the severity of fires in the region (Lounela, 2019). The most recent effort is the peatland restoration project (Badan Restorasi Gambut ‐ BRG), launched in 2016 as part of the “Presidential Instruction” (Detiknews 13.1.2016). However, rehabilitation efforts have largely been unsuccessful as corruption, poor governance, and a lack of accountability means many of these state‐wide regulations and restoration schemes are flouted or flagrantly ignored (Lounela, 2015, 2019, 2021; Mulyani & Jepson, 2015). With conflicting interests between different ministries, business actors, and local groups, limited resources, and few means of enforcing state‐wide legislation, there is a need to reconsider the current fire prevention methods and policies. Therefore, it is crucial to understand the main drivers of fire distribution and the conditions of initiation to better inform future fire mitigation efforts, and prevent ineffective, harmful, or costly prevention schemes that may, in the worst case, exacerbate peatland vulnerabilities. Existing studies have developed relationships that characterize the main drivers of fires within single years, either concentrating on one exceptional instance (such as 1997, or 2015) (Miettinen et al., 2017; Sumarga, 2017), or have attempted to quantify the significance of one type of influence, either anthropogenic, land use and land cover change (Miettinen, Shi, et al., 2012; Prayoto et al., 2017), meteorological (precipitation and El Niño) (Field et al., 2016; van der Werf et al., 2008), or mineralogical (peat depth, extent, etc.) (Cattau et al., 2016; Putra, 2011; Vetrita & Cochrane, 2019). However, no study has yet considered a wide array of drivers of multiple types, in combination across multiple fire seasons.

To this end, we aimed to develop a fire susceptibility model using machine learning that characterizes the relationships between key predictor variables and the distribution of historic fire locations to ascertain the relative importance of each in controlling the spread of fire. Our study is thus the first analysis in the region to encompass a wide range of driving factors within a single model that captures the inter‐annual variation as well as the spatial distribution of peatland fires. We included spatially explicit predictor variables within our analysis that characterize the climatic, anthropogenic, and environmental factors affecting fire distribution. We then used historic moderate resolution imaging spectroradiometer (MODIS) and visible infrared imaging radiometer suite (VIIRS) fire hotspot data as the dependent variable for fitting an extreme gradient boosted (XGBoost) model for use as a binary classifier identifying each location (pixel) within the landscape as either fire or not‐fire (1 or 0).

### 1.1 Study Area

**Figure 1:**
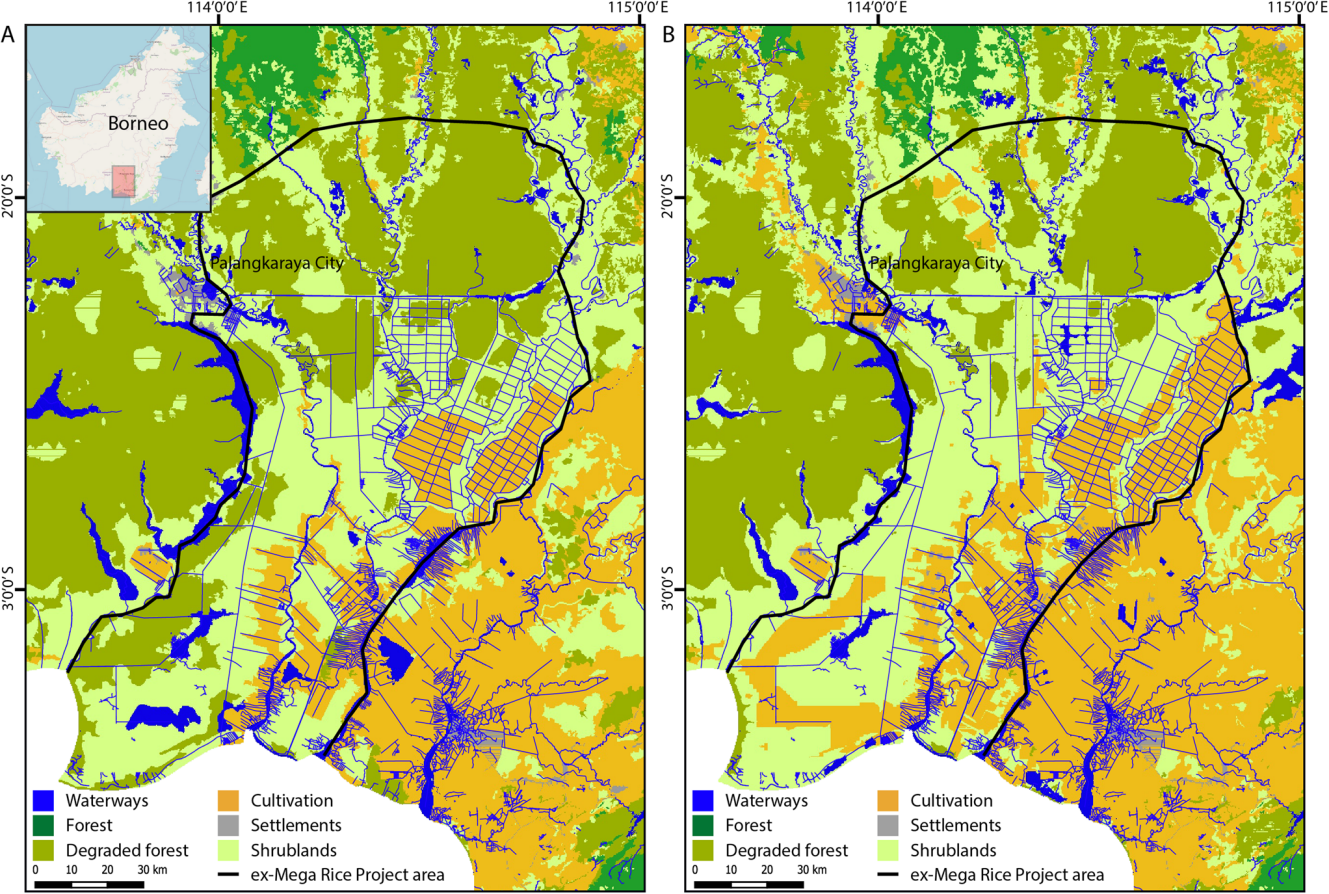
Ex‐Mega Rice Project area of Central Kalimantan on the island of Borneo. Land cover designations as of (a) 2000, and (b) 2015.

The ex‐Mega Rice Project (EMRP) of central Kalimantan covers an area of roughly 1 million hectares and lies to the south east of the Central Kalimantan Provincial capital of Palangkaraya, between the Sebangau and Barito rivers (Figure 1). Between January 1996 and July 1998, over 4,000 km of drainage canals were dug to lower the water table of the predominantly peat‐land area, and large areas were cleared of dense swamp forest for rice cultivation (Putra, 2011; Putra et al., 2008). However, the MRP was a failure as the infertile and acidic peatland was unable to support more than a few rice crop cycles without resorting to the traditional small‐scale cultivation practices of burning the top layer, which are not suitable for industrial scale application.

### 1.2 Climate and Fire Season

The population of the EMRP area consists of what is generically called Dayak groups, including different subgroups such as the Ngaju, Ot Danum, and Maanyan, and transmigrants mainly from Java and Bali. Local indigenous groups have traditionally practiced swidden cultivation and horticulture, hunting and gathering, while fishing provides an important source of food. In recent decades, many have shifted to cash cropping, mining, and timber logging, whilst continuing at the same time the old subsistence practices (Jewitt et al., 2014; Lounela, 2021). A large migrant population has settled in the area since the 1960s, mainly from Java and Bali, to work the rice fields. The Mega Rice Project brought another big wave of transmigrants to the region, but many of them now have to contend with poor agricultural yields and face some of the highest poverty rates in the region, forcing many to relocate back to their home islands and sell land to large companies (Goldstein, 2020). In 2005, poverty rates reached 36% in the local settlements and as high as 75% in some transmigrant villages (de Groot, 2008), which are far higher than the average for the wider Central Kalimantan province of 9.4% in 2007 (Bidang Statistik Sosial, 2012). Today, the EMRP area is a mosaic of subsistence rice farming, rubber plantations and fruit gardens, *Acacia mangium* stands for paper production, and large oil‐palm plantations all interspersed with scrubland and degraded forests. Although the peatland fires that recur each year are mainly anthropogenic in origin, the precise mechanism of ignition is unclear. They are most likely started by either small‐scale farmers for land‐clearance or to renew the topsoil fertility, or by private companies and government agencies to expand agricultural land into forest stands (Cattau et al., 2016; Sumarga, 2017; Vetrita & Cochrane, 2019).

**Figure 2:**
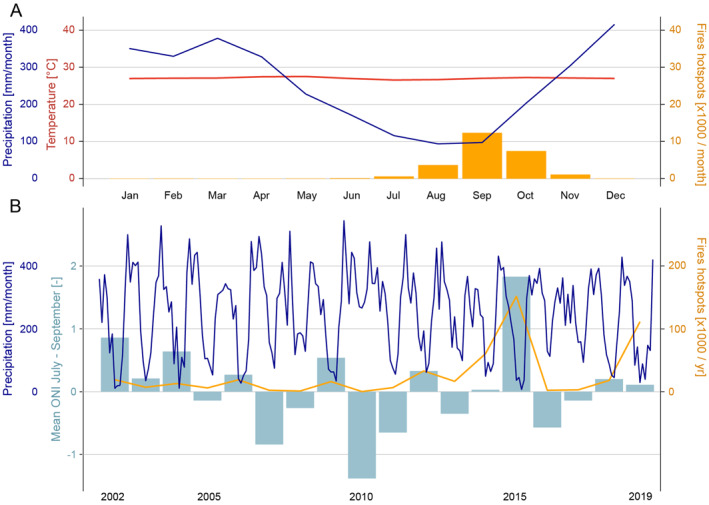
Climate and fire attributes of the study area. (a) Mean monthly precipitation (blue line), and temperature (red line) between 2002 and 2019 for the wider study area as bounded in Figure 1, as well as mean number of fire hotspots (bars) taken across the same years (2002–2019). (b) The monthly precipitation totals averaged across the study area shown in Figure 1 for years 2002–2019 (blue line), the mean ONI for July–September (early fire season) from 2002 to 2019 (bars), and the total number of fire hotspots detected by MODIS and VIIRS satellite sensors for each year 2002–2019 (orange line).

Measurements of mean monthly precipitation and temperature (Huffman et al., 2018; NOAA, 2021) taken across the wider study area as bounded by Figure 1 (2002–2019) show a distinct dry (May–October) and wet season (November–April), yet a consistent mean temperature (Figure 2a). The fire season is most pronounced from August to October, with very few fires occurring before July or after November (Figure 2a). As well as intra‐annual variation, there are significant inter‐annual differences between the number of fire hotspots detected by satellite sensors, with years such as 2010 recording only 100's of instances, whilst years such as 2015 may record upward of 150,000 hotspots (Figure 2b). Much of this inter‐annual variability can be attributed to the El Niño Southern Oscillation, which affects the spatial and temporal distribution of extreme precipitation events in the region, and so greatly impacts the distribution of fires. El Niño years exhibit greater fire prevalence, whilst La Niña years exhibit diminished fire prevalence (Fanin & van der Werf, 2017; Spessa et al., 2015; Supari et al., 2018).

## 2 Data and Methods

### 2.1 Data Sources and Pre‐Processing

Within our analysis, we included the spatially explicit predictor variables listed in Table 1. Before constructing the fire prevalence model, many of the predictor variables and the dependent variable (fire distribution) required pre‐processing to filter the data for quality control, and/or to convert to the correct format.

**Table 1:** Model Input Data Sources, Citation, Original Resolution, and Date Ranges

Data set (variable)	Data source	Citation	Resolution	Temporal coverage
MODIS fire hotspot (fire distribution)	MODIS product MCD14DL (collection 6)	Giglio et al. (2016)	1 km	2001–2019
VIIRS fire hotspot (fire distribution)	Product VNP14IMGTDL_NRT	Schroeder et al. (2014)	375 m	2012–2019
NDVI and EVI (vegetation indices)	MODIS product MOD13Q1	Running et al. (2017)	230 m	2001–2019
ET and PET (ET:PET ratio)	MODIS product MOD16A2	Didan (2015)	500 m	2001–2019
SPEI (3 and 12 months)	Calculated from ET/PET and NASA's GPM rainfall data	Vicente‐Serrano et al. (2010); Huffman et al. (2018)	0.1 deg	2001–2019
Distance to roads	OpenStreetMap	OSM (2018)	250 m	2015
Distance to canals	OpenStreetMap	OSM (2018)	250 m	2015
Distance to settlements	OpenStreetMap	OSM (2018)	250 m	2015
Indonesia Ministry of Forestry land cover (land‐ cover type, forest clearance index)	National Forest Monitoring System (NFMS)	MoF (2016)	125 m	1996, 2000, 2003, 2006, 2009, 2011, 2012, 2013, 2015
Oceanic Niño Index (ONI)	NOAA/National Weather Service	NOAA (2019)	NA	2001–2019
Elevation/slope/aspect	SRTM	Jarvis et al. (2008)	90 m	2000
Peat depth	CIFOR wetland project	Gumbricht et al. (2017)	231 m	2017

#### 2.1.1 Fire Hot Spots

Both moderate resolution imaging spectroradiometer (MODIS) and visible infrared imaging radiometer suite (VIIRS) fire hotspot data represent positive instances of fire occurrences as single points at the center of grid‐squares. Only MODIS fire hotspot data is available until 2012, after which time we use both MODIS and VIIRS data. Each hotspot within the datasets indicates at least one fire detection within the grid‐square of its respective resolution (1 km or 375 m), and as such, do not correspond to precise locations. To prepare the data for input as the dependent variable within the model, we first collate all fire hotspots (both MODIS and VIIRS) for each year and apply a quality control criterion to separate hotspots into two subgroups of high and low confidence occurrences as follows. We overlaid a 500 m grid and classified hotspots within grid cells containing only single hotspots or hotspots separated from all others by more than 30‐day as low‐confidence occurrences, and hotspots within cells that contain multiple instances of fire within 30‐day periods as high‐confidence occurrences. This ensures multiple instances of fire detection within close proximity and short duration within the high‐confidence subgroup, reducing the likelihood of false positives. Having defined these two subgroups, we then created a 500 m buffer (the maximum distance a MODIS hotspot is located from the corresponding ground location) around all points in each subgroup and dissolved the buffers to partition the study area into three spaces: high‐confidence fire occurrence space, low‐confidence fire occurrence space, and high‐confidence non‐occurrence space (outside both buffer areas). We then take random sample points from within the high‐confidence fire occurrence and high‐confidence non‐occurrence spaces to use as positive and negative training data respectively.

To estimate the impact of cloud cover on the inter‐annual variability of fire hotspot detection, we calculated the average number of cloud days within the fire season (July–October) across our study area for all years (2002–2019). Using the state_1 km band in the daily MODIS terra product (MOD09GA version 6), which classifies each pixel as either “no cloud,” “cloud,” “mixed,” or “unknown,” we counted the number of ‘cloud’ or ‘mixed’ designations for each pixel for the fire season each year. We then took the average of these values across the whole study area outlined in Figure 1 to give the average number of cloud days within the fire season for each year.

#### 2.1.2 Land Cover

The Indonesian Ministry of Forestry land cover map originally distinguished between 25 different land cover types, which we grouped to a reduced set of 8 (Table S1 in the Supporting Information S1), comprising of “Primary and secondary dry forest,” “Swamp forest,” “Swamp scrubland,” “Scrubland, Transition, and bare land,” “Riceland,” “Plantation,” “Settlements,” “Water and Cloud.”

The land cover map was converted from the original vector shapefile to raster format, with an option within the model structure to input the data either as a single raster image with values corresponding to land cover designations, or as multiple rasters each depicting the distribution of a single land cover type as a binary (0 or 1) image.

In addition to land‐cover inputs, we tracked changes in forest extent and generated an index to denote time since forest clearing/growth. The 8 land cover designations were re‐grouped into forest (1) and non‐forest (0). We then generated a set of loss/gain rasters, assigning a pixel that changes from forest to non‐forest (i.e., 1 to 0) between time‐steps an index of −10, and a pixel that changes from non‐forest to forest (0–1) an index of 10. For each subsequent time step that it remains its new designation, the index reduces by one toward zero indicating a reduction in the index severity. Therefore, an area cleared of forest between land cover maps 2000 and 2003 will be assigned the index value −10 for years 2003–2005, which reduces in magnitude by 1 each subsequent map iteration it remains non‐forest, so that by 2015 it has the designation −4.

#### 2.1.3 Vegetation Indices

The ET, PET, NDVI, and EVI are all pre‐prepared MODIS products, which required no pre‐processing other than to infill missing values with the mean of surrounding pixel values within a 5 × 5 window, and converting the shorter time frames (8 and 16 days respectively) to monthly averages. Rather than use absolute values, we use the ratio of ET to PET as a model input, as this is a better proxy for the soil moisture deficiency of an area.

For all vegetation indices, we included the option of normalizing the values by a reference area, which can be added as a shapefile model input. We identified an area of primary forest to use as a reference input to indicate the state of all pixels relative to an assumed norm. Normalized variables are then reported as the ratio of the measured metric at each location against the same metric at this reference area.

#### 2.1.4 SPEI

We combined MODIS ET and PET products with NASA's global precipitation measurement (GPM) rainfall data (Huffman et al., 2018) to calculate the 3‐ and 12‐month standardized precipitation‐evapotranspiration index (SPEI) from the beginning of each month for all years (2001–2019). Initially we bilinearly interpolated the rainfall data (0.1°) to the finer resolution of the ET/PET data (500m), then performed the calculation pixelwise.

#### 2.1.5 Proximity to Anthropogenic Factors

The distance to roads, canals, and settlement rasters were derived from OpenStreetMap data as the Euclidean distance to nearest feature in 250 m resolution. We have not accounted for development of roads and settlements over time, and the historic differences are one source of error. However, the vast majority of canals were built during the MRP initiation between 1996–1998 and so should not account for much error.

#### 2.1.6 Oceanic Niño Index

The ONI is one measure of the El Niño‐Southern Oscillation taking ocean temperature anomalies as rolling three‐month averages. For each year, we use just one value corresponding to the early fire season (July, August, and September) (Figure 2), generating a coarse raster image with this single value as the model input.

#### 2.1.7 Elevation (SRTM)

From the SRTM 90 m elevation raster, we derived local slope and aspect (Ritter, 1987). We then reduced the spatial resolution of all three variables to 250 m for processing efficiency.

#### 2.1.8 Cross Year Normalization

Before using the predictor variables as training data, we normalize each across all years within the training data set, such that:

$$V_{\text{norm}}=\frac{V-V_{\text{min}}}{V_{\text{max}}-V_{\text{min}}}$$

where $V_{\text{norm}}$ is the normalized version of the predictor variable V, $V_{\text{max}}$ is the maximum value within the training data set across all years (2002–2019), and $V_{\text{min}}$ is the minimum value within the training data set across all years.

#### 2.1.9 Fire Season Data Inclusion

The aim of our investigation is to construct a model that faithfully replicates the distribution of fire occurrence across the ex‐MRP area based on the spatial and temporal distribution of driving variables to better understand the conditions that cause fire events. Therefore, we have included data that gives information about the condition prior to the fire season, and data from the time spanning fire occurrences, to understand the relationship between the two. To fully explore the impact of these inclusions, we have constructed three separate models, one that includes both pre‐fire‐season and fire‐season data (model M_pre&fire), one that includes only fire‐season data (model M_fire), and one that includes only pre‐fire‐season data (model M_pre). Fire season data was taken as an average over the period August‐October and pre‐fire‐season data is taken as an average over May‐July. For the pre‐fire‐season model (M_pre), we also included the additional predictor variables that indicate the change in vegetation indices between pre‐fire‐season and August (Aug–pre‐fs), for ET:PET ratio, EVI, and NDVI.

### 2.2 Model Structure and Application

The XGBoost algorithm compiles an ensemble of decision trees generated from subsets of input predictor variables based on a tree boosting selection criteria (Chen & Guestrin, 2016). It has demonstrated improvements in the application of land‐cover classification problems over other gradient boost and standard random forest (RF) algorithms (Chen & Guestrin, 2016; Man et al., 2018; Memon et al., 2019). The branching of individual decision trees is done by splitting the training data set according to a property of one of the predictor variables (i.e., above or below a threshold, belongs to one classification or doesn't, etc.), and repeated for each predictor variable in the training data set to form a tree. At the end of each branching pathway along the decision tree, the training samples belonging to that pathway end‐state (i.e., have been split along the pathway) have designations of either fire or non‐fire attached. The proportion of fire designations to the sum of fire and non‐fire designations denotes the probability of a fire occurring at a location with that subset of predictor‐variable properties collected by this individual decision tree. Applying the model over a grid covering the study area then consists of sampling the predictor variable properties at each grid cell, routing them down all decision trees within the ensemble, and finally averaging the probability of fire predicted at each pathway end‐state.

**Figure 3:**
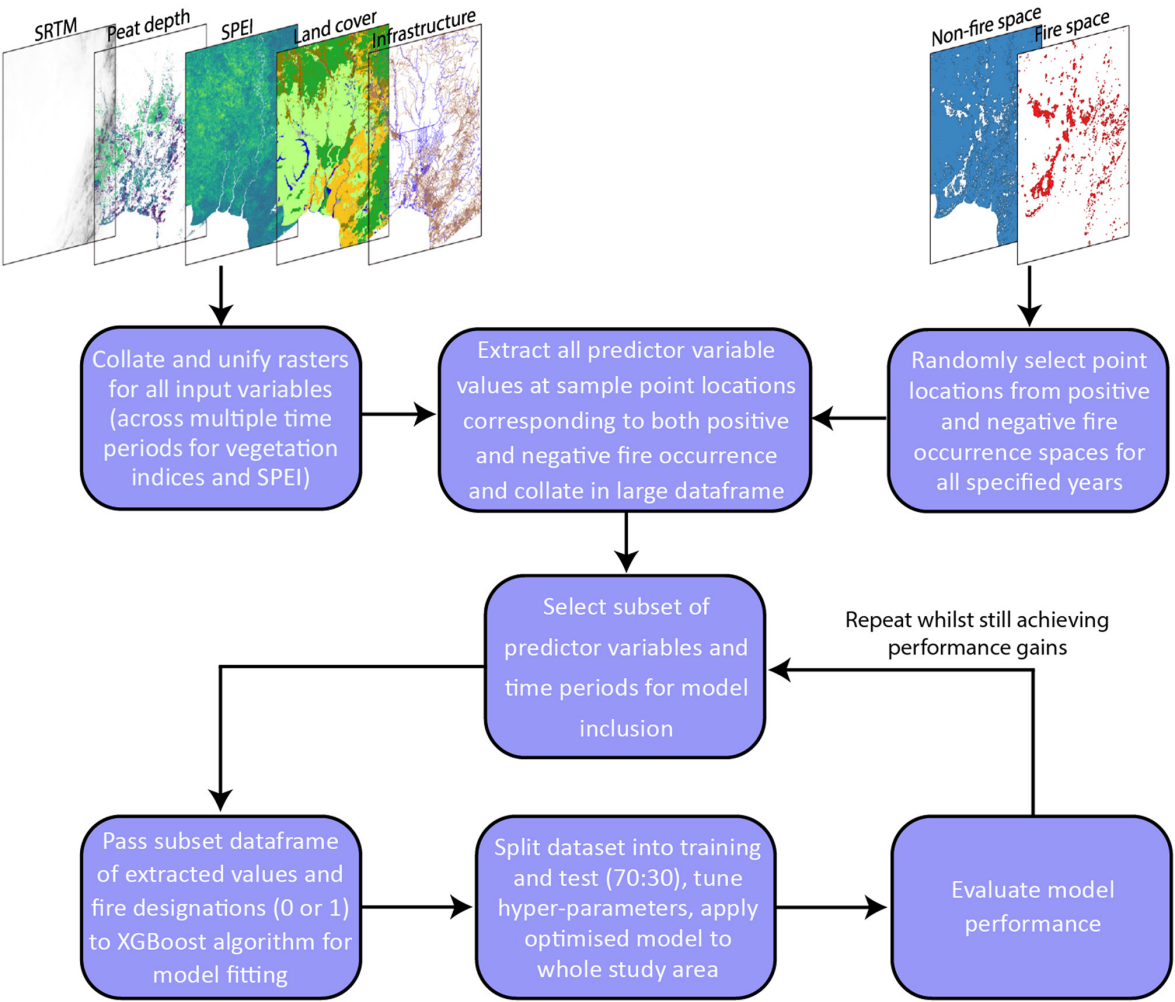
Model flow diagram outlining processes and feedbacks.

The initial stage of model application is to spatially align and resample all predictor variable input rasters to the same extent and resolution before randomly selecting extraction points from within the fire‐ and non‐fire‐occurrence spaces for each year (2002–2019). The values for each predictor variable as well as the fire designation at the sample location are compiled into a large data set. From this large data set, subsets of predictor variables can be selected for model training and comparison to optimize the input variables based on model performance. For the training of each model the XGBoost hyper‐parameters are tuned by splitting the training data set into train and test portions (70:30) and using a grid search technique, whereby model performance for every combination of hyperparameters within a given range are evaluated to form a grid, from which the best combination is selected. The optimized model derived from each subset of input variables was then validated and compared to find the best performing model (the procedure is outlined in Figure 3).

### 2.3 Validation

We validated the model year on year by assessing the accuracy, precision, and recall of probability estimates of fire occurrence across all locations (pixels) within the MRP bounds against a density map of fire hotspot data taken from all fire hotspots (without the quality control applied to defining fire‐occurrence spaces). Both the model probability estimate, and the density of fire hotspots require a threshold above which a location is designated as a positive fire occurrence. To evaluate model performances across all years (2002–2019), we examined the precision, accuracy, and recall using a constant probability estimate threshold of 0.75 for a positive fire designation, and took the 90th percentile of the fire hot‐spot density distribution of each year as the threshold for fire occurrence at a location.

Accuracy=(TP+TN)/(TP+TN+FP+FN)

Precision=(TP)/(TP+FP)

Recall=(TP)/(TP+FN)

where TP is true positive, TN is true negative, FP is false positive, and FN is false negative. These proportions are then converted into percentages by multiplying by 100.

Then, for the example year of 2018, we evaluated the model performance over a range of thresholds for both probability (0.5–0.9) and density (scaled by total number of hotspots) to map out a validation space between the three metrics of accuracy, precision, and recall.

## 3 Results

Initially we evaluated the overall performance of the three models (M_pre&fire, M_fire, and M_pre) averaged across the EMRP Area (as shown in Figure 1), across all years (2002–2019), comparing accuracy, precision, and recall. We then evaluated the relative importance of each driving variable within the model structures to ascertain the most influential factors of inducing and spreading peatland fires, as well as characterizing the differences between models. Lastly, we analyzed the example year of 2018 in more detail to highlight the differences between models and illustrate shortcomings of the models' predictive capabilities. Year 2018 was chosen as the example year as there were widespread fire occurrences throughout the fire season, but it was not an exceptional year (such as 2015 or 2019). Finally, we assessed the importance of each predictive variable in the three model setups.

### 3.1 Model Evaluation Across All Years

**Figure 4:**
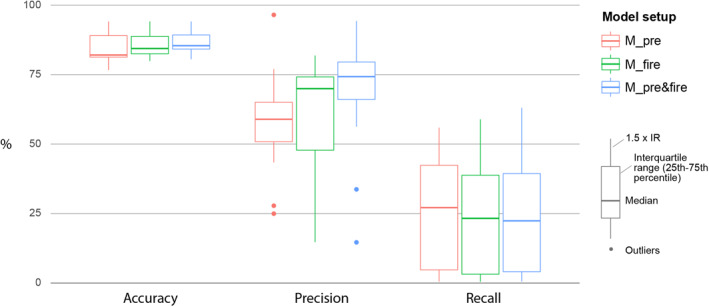
Boxplots showing accuracy, precision, and recall for M_pre (model containing pre‐fire season data) (red), M_fire (model containing fire season data) (green), and M_pre&fire (model containing both pre‐fire season and fire season data) (blue) for years 2002–2019 (see Figure S1 in the Supporting Information S1 for yearly results). Boxes denote the interquartile range (25th to 75th percentiles), whiskers denote values within 1.5 times the interquartile range (IQR) from the upper/lower limit of the IQR, and dots mark outliers beyond that scope. Accuracy is defined as the percentage of estimates (positive and negative) that matched observations, precision is the percentage of positive estimates that matched observations, and recall is the percentage of total fires the model correctly identified. These metrics were taken as mean values from across the EMRP area as shown in Figure 1.

The model that includes both pre‐fire and fire season information (M_pre&fire) consistently performs well in terms of both accuracy and precision, with the only exceptions being the years of 2008 and 2016, which are classified as outliers for the precision metric, and can be seen as dots in Figure 4 (see Figure S1 in the Supporting Information S1 for yearly metric scores). The recall metric, however, scores low for all years, only exceeding 50% once in 2015. Recall denotes the proportion of observed fire instances that the model accurately captured, meaning that the model captured less than 50% of all reported fires in all years except 2015. That accuracy and precision score highly whilst recall is much lower, suggests that the model fails to register large areas where fires may occur, but we can have a high degree of confidence in the model predictions for areas that it does designate as fire. The model that includes only fire season data (M_fire) performs similarly to M_pre&fire, though the precision is reduced in nearly all years, whilst accuracy and recall are almost unchanged (Figure 4). The model that includes only data before the fire seasons starts (M_pre) performs the worst, with a significant reduction in the median value for precision, and although the recall metric is slightly improved compared to M_pre&fire and M_fire in several instances, it still performs poorly (Figure 4).

To determine how model M_pre&fire may perform when applied to years not included in training the model, we derived a new version of the M_pre&fire model based on the same input variables and hyper‐parameters as the original for each year between 2002‐2019, but excluding one year's data from the training set. We then applied the model to the excluded year and evaluated the model performance.

**Figure 5:**
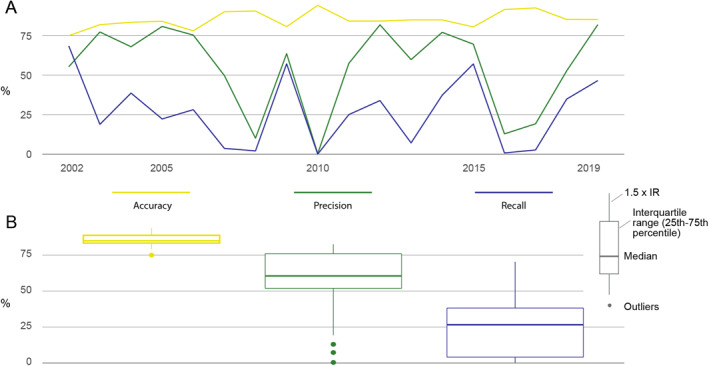
Results showing accuracy, precision, and recall for each year corresponding to a separate model that follows the M_pre&fire setup, but where each year's model was excluded from the training data set. (a) Recall (blue line), Precision (green line), and Accuracy (yellow line) for years 2002–2019. (b) Boxplot showing median, interquartile range, whiskers, and outliers for each metric shown in (a). Accuracy is the percentage of estimates (positive and negative) that matched observations, precision is the percentage of positive estimates that matched observations, and recall is the percentage of total fires the model correctly identified.

Except for years 2008, 2010, 2016, and 2017; model precision is consistent with the original evaluation of model M_pre&fire (Figure 5 and Figure 4a). These notable exceptions coincide with years that recorded very few fire hot spots, with 2010 recording just 242 fire occurrences compared to the average of 27,013 occurrences (Figure 2b). Not including these exceptional years, the average model precision score is 68%, which is comparable to the original evaluation of model M_pre&fire, which had an average precision score of 71%.

### 3.2 Year 2018 Results as an Example Year

**Figure 6:**
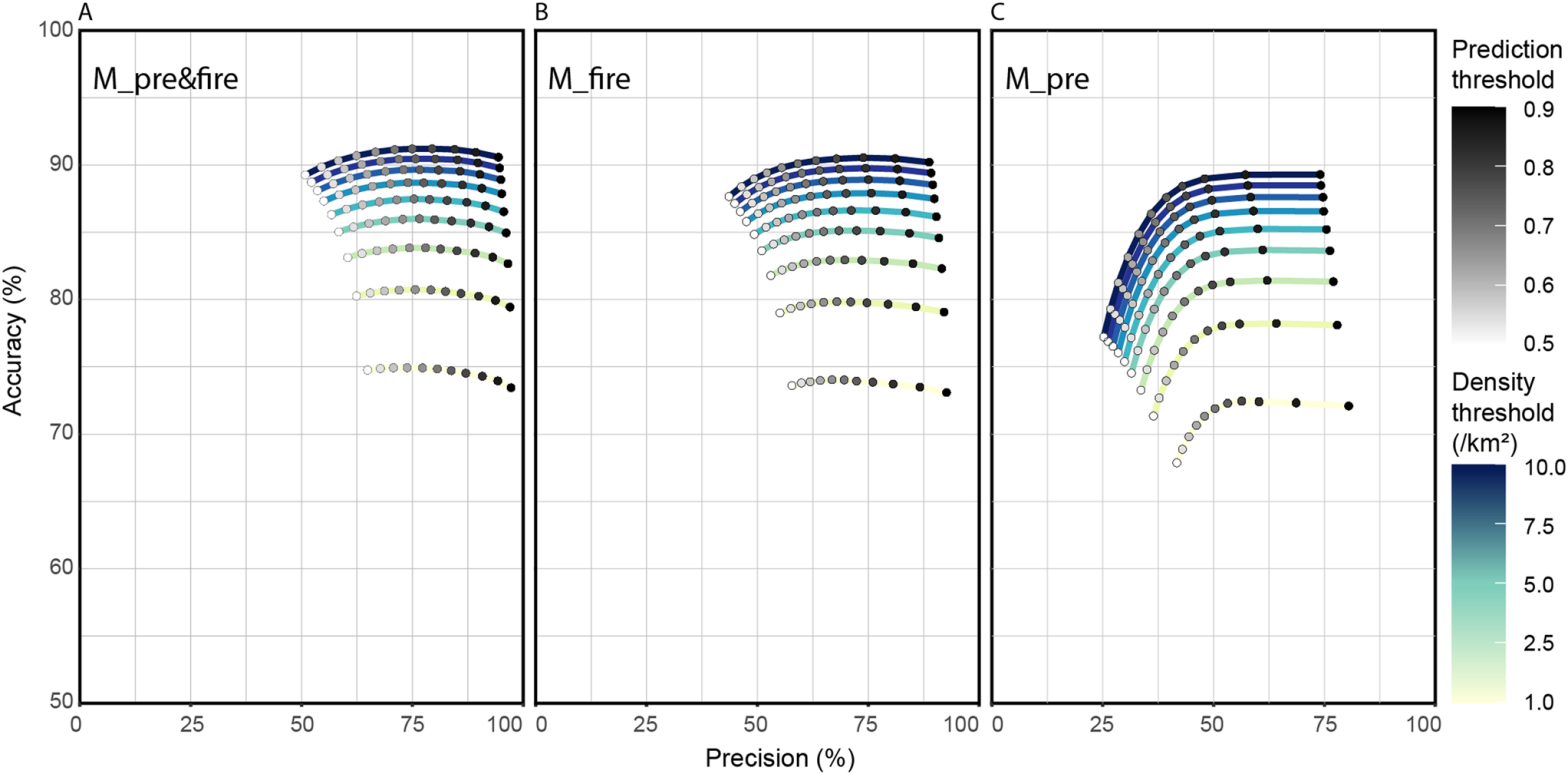
Accuracy and precision scores for varying prediction and fire density thresholds. (a) Model M_pre&fire, (b) model M_fire, and (c) model M_pre. Accuracy is the percentage of estimates (positive and negative) that matched observations, and precision is the percentage of positive estimates that matched observations.

Mapping out a model space by varying the prediction threshold for fire occurrence (between 0.5 and 0.9: denoted by the grayscale dots) and the observed fire hotspot density threshold for denoting fire occurrence at a location (between 1 and 10 hotspots/km^2^: denoted by the color range of the lines), illustrates the range of model performance for each of the three models for the example year of 2018 (Figure 6).

Models M_pre&fire and M_fire perform better in terms of both accuracy and precision (Figures 6a and 6b). For 2018, model M_pre&fire's precision score ranges from 51%–98%, and accuracy ranges from 74%–92% depending on the prediction threshold and density threshold designations. These metrics decrease slightly for model M_fire (precision: 39%–93%, accuracy: 73%–91%), but significantly decrease for model M_pre (precision: 25%–78%, accuracy: 67%–89%).

**Figure 7:**
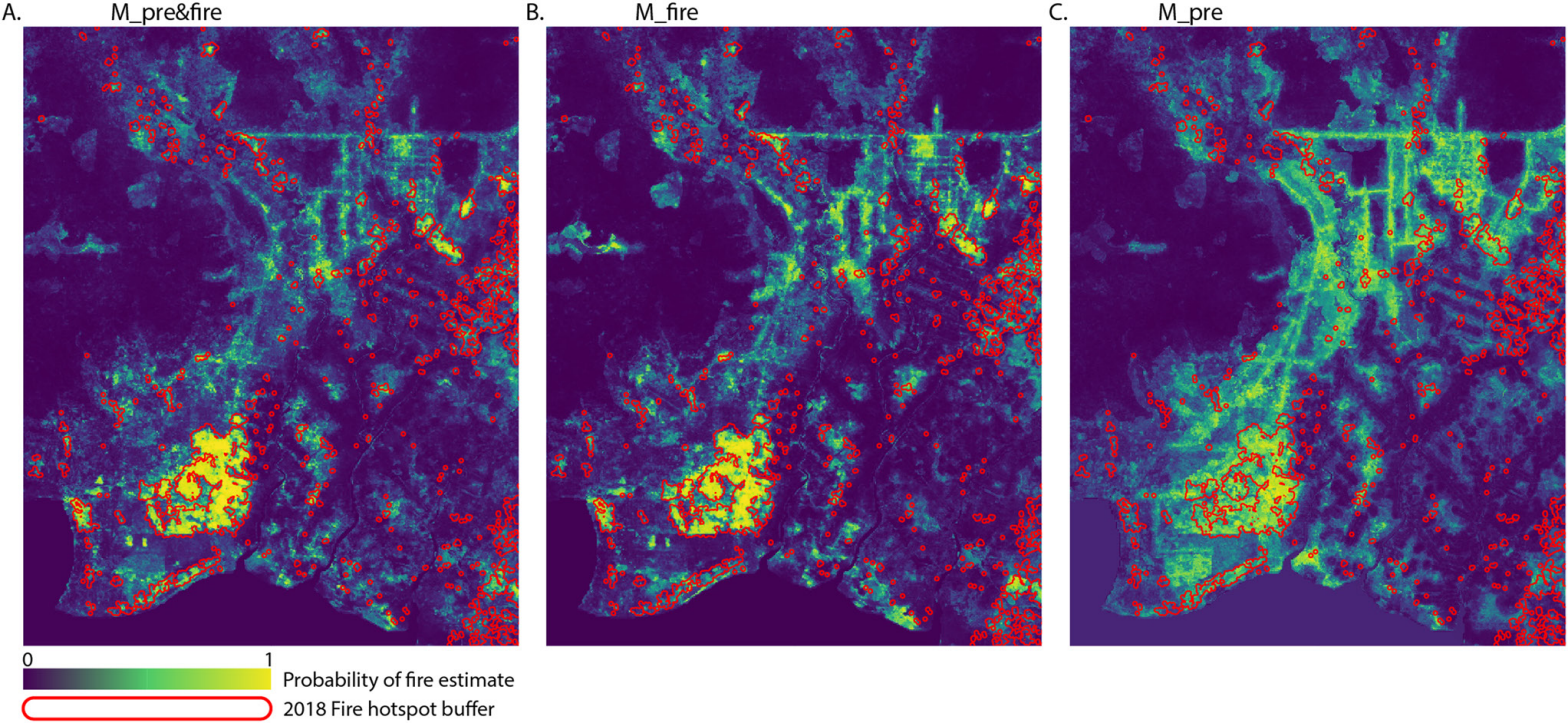
Model predictions of fire occurrence as a probability (0–1) for 2018 and observed distribution of fire hotspot data displayed as combined buffer zones (500 m) around confirmed instances (red outlines) for (a) M_pre&fire, (b) M_fire, and (c) M_pre.

The differences in model composition can clearly be seen in the distribution of predictions observable in the example year of 2018 (Figure 7). Model outputs from M_pre&fire and M_fire show a distribution of fire estimation that is much more closely aligned to the observations of fire hot spots, with relatively small areas of high probability located throughout the region, not just within areas of high anthropogenic activity. By contrast, model M_pre more uniformly distributes the likelihood of fire occurrence along infrastructural lines, and assigns a relatively consistent probability of fire‐occurrence that covers the area with a high concentration of canals, roads, and settlements.

### 3.3 Driving Variables

Having established the relative performance of each of the three models, comparing the difference in model structures and relative importance of each of the predictor variables should allow us to better understand the conditions needed to initiate and sustain fires.

**Figure 8:**
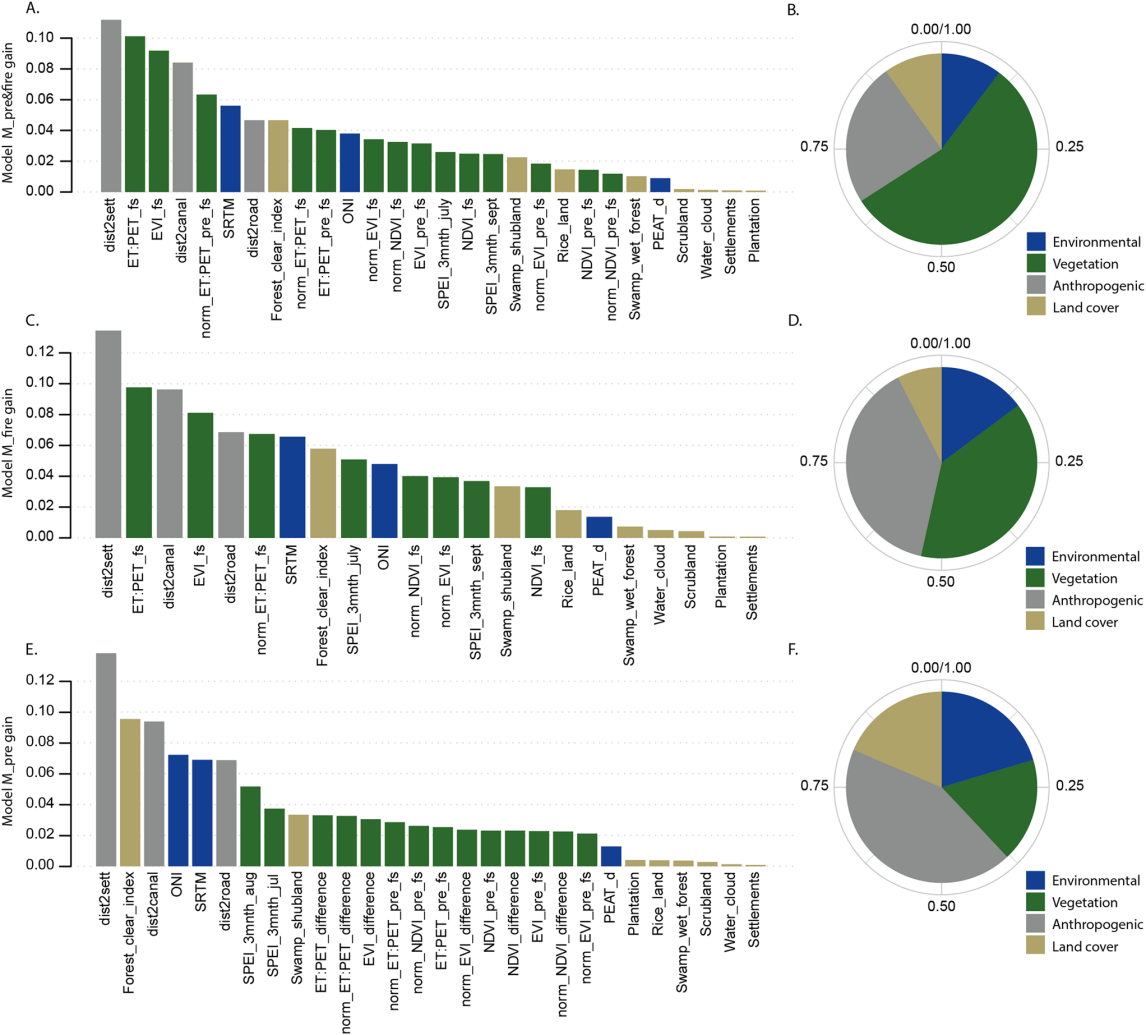
Importance of drivers in each model setup as a proportion of the total importance summing to one. (a and b) The proportional importance of each predictor variable as well as group of predictor variables within model M_pre&fire. (c and d) Proportional importance for model M_fire. (e and f) Proportional importance for model M_pre. A description of each of the variable names can be found in the supplementary, Table S2 in the Supporting Information S1.

The anthropogenic factor of ‘distance to settlements’ is consistently weighted the highest importance for denoting whether a location is categorized as fire or non‐fire for all three models, followed closely by “distance to canals.” Where the fire‐season data is included in the model (models M_pre&fire and M_fire), the fire‐season ET:PET ratio and EVI are also very strong predictors (see details of data in Table 1). When fire‐season data is not included (model M_pre), the forest clearance index (Forest_clear_index), and ONI are more prominent indicators (Figure 8c). These structural differences between the models are illustrated in the respective proportional influence of environmental factors (SRTM, ONI, Peat depth), anthropogenic proximity, vegetation indices, and land cover impacts within the top 10 weighted predictor variables. For model M_pre&fire, vegetation indices constitute around half of the weighted gain (internal model metric for denoting importance) within the top 10 predictor variables (Figure 8a). The proportional weighting is equally split within model M_fire between the anthropogenic proximity indicators, and vegetation indices (Figure 8b), and in model M_pre, it is the anthropogenic proximities that are dominant, with the contribution from the land cover impact and environmental factors increasing to roughly the same proportion as the vegetation indices (Figure 8c). This accounts for the differences in probability distributions observable in 2018 (Figure 7), showing model M_pre uniformly assigning higher probabilities across areas in close proximity to anthropogenic influences with little distinction within those areas.

## 4 Discussion

### 4.1 Interpretation of Model Performance and Structure

The main model (M_pre&fire) performs well across all years with sufficient fire hotspots for a robust validation, consistently scoring highly in accuracy and precision (>75% and >65% respectively) (Figure 5). This indicates that areas designated as fires are, for the most part, correctly identified. However, the model does not capture a large proportion of all observed fire occurrences, with the recall metric scoring much lower. Our results demonstrate the anthropogenic dependence of extreme fire events in the region, with the distance to settlements and distance to canals consistently weighted as some of the most important driving factors within the model structure (Figure 8). In combination, the vegetation indices were the strongest indicators of fire prevalence–especially during the fire‐season months of August to October, which can be seen in the relative dominance of the vegetation variables in models M_pre&fire and M_fire (Figures 8a and 8b). That these vegetation predictor variables are not as dominant in model M_pre suggests that they are strong predictors of fire locations in later months due to the change in the vegetation indices being brought about by fires, rather than the indices themselves being precursors or indicators of where fires will happen in the future. In addition, the forest clearance index (time since forest clearing) and ONI consistently ranked highly, suggesting that these two factors are important precursors to fires. The ONI in particular is an important consideration as it is the only “global” predictor variable, in that it remains constant across space and changes only through time, as is shown by it's relative importance especially in model M_pre, suggesting it may be an important consideration in any predictive model. Capturing the impact of El Niño and La Niña years on the prevalence of fires in any one year is paramount to replicating the inter‐annual variation within the model, as the Southern temperature oscillation has been shown to strongly control fire activity and impacting other driving variables within the model structure, such as precipitation patterns (SPEI 3 months variable) and vegetation indices (Spessa et al., 2015; Supari et al., 2018).

Comparing the composition and performance of models M_fire and M_pre show that the conditions necessary to propagate large fire events exist uniformly across areas where human activity frequently occurs, but the precise location of fire initiation is not predetermined prior to the fire season by the factors that we have included in our analysis. Nevertheless, distance to settlements and distance to canals are still two of the largest influences on model determination in the fire‐season model. Distance to settlements is a proxy for human access to an area, and distance to canals is related both to the hydrological condition of the underlying peatland (as the water table level is lower near the canals) and to human access (as canals are used as waterways). That the proxy for human activity is the main indicator of fire susceptibility aligns with the assertion that most fires are anthropogenic in their origin; this most probably includes large‐ and small‐scale agricultural or plantation activities and land clearing initiatives to expand existing plantations (Sumarga, 2017; Vetrita & Cochrane, 2019). Another important finding is that the positioning of the forest clearance index and land cover classifications within the rankings of model predictors indicates that the type of successional land cover to follow forest clearing is less important than the time since clearing. That the forest clearance index was consistently more important than the highest ranking land cover classification (swamp scrubland, i.e., cleared areas of swamp forest that had not been cultivated) suggests that preserving forest is much more effective in preventing fire than effectively managing the land after forest removal.

The detailed results for the 2018 example year demonstrate that, without additional information, the precise locations of fire occurrences are not possible to predict prior to the fire season commencement with the data and methodologies used in this study. M_pre's estimation represents the spatial area most prone to fire outbreaks based on the infrastructural and land development patterning at the time (Figure 7c), and the relatively uniform probability assignment represents the fire‐seasons propensity to burn based on the wide‐scale environmental factors in 2018, such as the ONI, amount of rainfall (SPEI index), etc. The exact location of fire instances within this general area of high susceptibility are most likely governed by human activity, and so not easily predictable. However, models M_pre&fire and M_fire are able to capture the precise location of fire instances as the fires leave an indication of their location within the vegetation indices of ET:PET ratio and EVI. Though these indices alone are not enough to detect fires consistently, as many areas throughout the region display ET:PET ratios and EVIs comparable to areas that have experienced fires. This is again demonstrated in the importance ranking of predictor variables within the model structures of M_pre&fire and M_fire, where anthropogenic factors are of the highest import.

Our results resonate with other studies that focus on fire distributions in the EMRP area and the surrounding Indonesian peatlands, showing anthropogenic drivers to be the major contributing factors both in terms of the distance to infrastructural variables, and the impact of disturbed land‐cover classifications (Cattau et al., 2016; Tan et al., 2020; Vetrita & Cochrane, 2019). In contrast to the findings of Prayoto et al. (2017), we found that proximity to roads and particularly canals to be a more significant factor than land‐cover type even within the swamp shrubland designations. The importance of canals in preserving the integrity of the peatland is highlighted in the study by Sinclair et al. (2020), who also note the impact on fire prevalence. However, our findings do support the assertion that unmanaged swamp shrubland that occupies areas of degraded or cleared swamp forest is the most susceptible to fires (Miettinen et al., 2017; Prayoto et al., 2017; Sumarga, 2017; Thoha et al., 2018; Vetrita & Cochrane, 2019). Our findings also agree with the assertion that plantation concessions are not significant sources of fires (Cattau et al., 2016; Prayoto et al., 2017), though as Tan et al. (2020) point out in their analysis, we have not accounted for the impact of plantations to the surrounding area, which may become evident if proximity to plantations were included. Contrary to the findings of Thoha et al. (2018) we found peat depth within peatlands to be a relatively weak indicator of fire prevalence, which may be an important consideration as there is currently legislation that prohibits certain practices only on deep peatland (>3 m) (Uda, 2020). Our most significant temporal variable is the ONI, a result that is supported by a number of studies that have found that El Niño events strongly affect the number of fires in Indonesian peatlands (Pan et al., 2018; Putra et al., 2008). Whilst our results echo many other studies in highlighting these key factors as controlling the intensity and distribution of fires in the region, our work unifies them into a single cohesive model to assess the relative weighting of each, and clearly demonstrates the dominance of proximity to anthropogenic infrastructure.

### 4.2 Model Limitations

The major limitation with our study is that the inclusion of fire season (August–October) vegetation indices in models M_pre&fire and M_fire carry with them evidence of past fire events, as the fires directly impact these indices. However, as we have already stated, these indices alone are not sufficient to precisely identify fire locations, as is evidenced by the fact that no vegetation index is the most significant model component. Yet this does change the dynamic of the model away from the development of a predictive tool and toward an explanatory descriptive methodology. Rather than use these spatial and temporal variables to predict the future distribution of fires, we have described the historic distribution of fires in terms of the major confining variables.

Whilst all three models manage to achieve high scores for accuracy and precision, recall is consistently low, only surpassing 50% in a couple of instances. This indicates that, although the models do a good job of identifying a specific type of fire occurrence, there may be other types of fires, driven by a different set of factors, that are not being captured within our analysis. An interesting aside may be to remove the fires detected by the current models, and repeat the training process on the reduced set of fire instances to gain insight into the factors most affecting these types of fires.

Testing the model M_pre&fire on years that were excluded from the training data set illustrates the impact of including multiple training samples from years with few fire instances. In 2008, 2010, 2016, and 2017, the inclusion of positive fire locations within the training data set structured the models to include decision trees that correctly identified a few exceptional instances when fires occurred in years where conditions were not suitable for widespread fires (Figure 5). Without these exceptional cases included in the model structure, the model failed to identify these small numbers of positive fire locations resulting in low precision scores. This level of dependency on a few positive instances of fire occurrence may cause overfitting in the model structure.

As with any study of this kind, a major limiting factor is the availability and quality of driving data. Most of the data we used was of good quality with high spatial and temporal resolution for our study area. However, the proximity to anthropogenic infrastructure taken from OpenStreetMap has no date associated with the data, it being a static appraisal of the situation circa 2018. Whilst this is unlikely to unduly affect the distance to canals (as the vast majority were dug in the late 1990s), roads and settlements that were developed later are incorrectly represented as existing throughout the entire study period. In addition, we used coarse resolution satellite images that may introduce uncertainties related to forest heterogeneity: a large pixel may include several types of forests that may influence the performance of the model. There may also be some uncertainty in using MODIS and VIIRS data to estimate the number of fire events. For example, due to short time periods, some fires may have started and ended between satellite overpasses, or may have been obscured due to cloud cover or heavy smoke. An analysis of the average number of cloud days during the fire season (July–October; 123 days) shows that cloud cover is fairly consistent across the years: median of 56 days, with the interquartile range spanning less than 10 days across the period 2002–2019, and the total range spanning from 43 to 67 days (Figure S2 in the Supporting Information S1). This is a stark contrast to the huge variability in the number of fire hotspots observed each year, which ranges from 240 to 151,000. As such, this strongly indicates that the differences in cloud cover alone do not explain the differences in fire hotspot observation. This is further confirmed in a poor correlation between the number of cloud days and fire hotpots in a year, with a coefficient of determination (*R* ^2^) of just 0.28. Missed observations from smoke cover, however, will not be consistent across years and will disproportionately affect detection in high fire years where smoke is more prevalent. This suggests that differences between fire occurrences in high fire years (such as 2015) and low fire years (such as 2010) may be underestimated.

### 4.3 The Human Element and Future Research

Whilst our model results highlight the relative importance of broad categories of drivers causing persistent fires in the EMRP area, we inevitably miss important distinctions and subtitles within these categories. For example, the distance to settlements variable consistently proved one of the most important of model determinants, but the EMRP is an assemblage of many different social groups with specific water‐related environmental practices, histories, and value orientations residing in locations where state and non‐state interventions have transformed the peat landscape (Lounela, 2021). For instance, the state may enhance restoration of the peat land while at the same time it supports industrial tree or oil palm plantations that demand drainage of the peat land (Lounela, 2021). This draining of peatland for cultivation will lower the groundwater level, reduce soil moisture content effectively drying out the peatland, and increase its susceptibility to burning. Furthermore, transmigration programmes–still going on in Central Kalimantan–have brought different groups of people in the region to participate in developing agricultural practices and industrial tree plantations, but because of the difficulties to make a living from the poor peatland, many have been forced to sell land to other actors (Goldstein, 2020). Beyond the factors included in our model, the increasing competition over land related to insecurity in terms of land tenure rights may also affect fires, since people can claim land rights and respond to industrial or agricultural expansions of the companies by using fire (Goldstein, 2020). Our current model is also incapable of including stakeholder actions within the analysis, which may not be attributed to any factor currently included in the model structure. Whilst our analysis indicates that areas designated as plantation concessions are not more susceptible to fires than other land cover classes, that does not preclude the actions of concession owners or corporations in contributing to the source of fires in the wider area. Such actions have been reported by localized studies, media outlets, as well as police investigations into the actions of 16 corporations surrounding the deliberate ignition of fires for clearing of forest to enable expansion of their own holdings (Dewi, 2019; Purnomo et al., 2019).

Understanding how these social drivers contribute to the likelihood of initiating the burning of an area may add another layer of complexity and explanatory power to the model structure. The distance to roads and canals could likewise be expanded upon to include their size and primary function, as these may again affect the likelihood of an initiation. These improvements would require a comprehensive understanding of the settlements, canals, and roads, as well as more thorough research into the driving principals behind peat fire ignition from a social perspective.

## 5 Conclusion

We developed a fire susceptibility model using machine learning (XGBoost random forests) that characterizes the relationships between key predictor variables and the historic distribution of fire locations in the EMRP area of Indonesian Borneo. The model was able to precisely determine areas that experienced fires when vegetation variables represented both pre‐fire season and fire season data (M_pre&fire), with precision scores consistently exceeding 70% for the study time period (2002–2019). However, the development of a predictive model with only pre‐fire season data included (M_pre) was less precise in determining areas experiencing fires (precision scores consistently 50%–75%). Recall metrics were low for all model setups, indicating that there may be fires driven by a different set of variables not characterized by the model. Our results demonstrate the anthropogenic origin and dependency of fires in the region, and suggest that the preservation of natural forest cover is of more importance to fire prevention than the subsequent management of the cleared land. Our model may be used to conduct similar analyses of the driving factors of fires in other parts of Southeast Asia or regions of the world, and contribute to a framework of mitigation tools to be employed by policy makers to help reduce the impact of widespread fires in the future. If suitable predictor variables and a stronger social component can be added to the model structure, it may be possible to use this methodology to develop functioning predictive models that would enable the region to better estimate where fires would concentrate in the future.

The inability of the predictive model setup (M_pre) to distinguish between areas that displayed equivalent infrastructural and environmental predictor values highlights the role of human agency in determining the precise location of initiation. This demonstrates the need for a more thorough understanding of the social drivers behind the practices of burning land and how they change spatially, temporarily, and in response to environmental drivers. The integration of these understandings into the model structure is required to estimate the likely locations of future initiations within areas designated as high risk due to the infrastructural and environmental factors identified by the existing model structure. This will necessitate a shift from the current large scale quantitative approach of numerical modeling to incorporate qualitative assessments of social variables and practices.

## Supporting information

Supporting Information S1
