# Supplementary material for: Identifying Key Drivers of Peatland Fires Across Kalimantan's Ex‐Mega Rice Project Using Machine Learning
Source: Earth Space Sci. 2021 Nov 24;8(12):e2021EA001873. doi: 10.1029/2021EA001873 (PMC9286596; doi:10.1029/2021EA001873)
Supplement: Supplementary file 1 — Supporting Information S1 [file ESS2-8-0-s001.docx]

## Supplementary Information for

Identifying key drivers of peatland fires across Kalimantan’s ex-Mega Rice Project using machine learning

Alexander J Horton^1^, Vili Virkki^1^, Anu Lounela^2^, Jukka Miettinen^3^, Sara Alibakhshi^1^, Matti Kummu^1^

^1^ Department of Built Environment, Aalto University, Espoo, Finland

^2^ Development Studies, Social and Cultural Anthropology, University of Helsinki, Finland

^3^ VTT Technical Research Centre of Finland Ltd., P.O. Box 1000, FI-02044 VTT, Finland

# Contents of this file

Tables S1 and S2, and Figures S1 and S2.

# Introduction

This supplementary information provides details of the original land cover classifications, the variable descriptions used within the analysis (as shown in Figure 8 of the manuscript), the year by year metric scores for each model taken as an average across the study area as summarised in Figure 4 of the manuscript, and an analysis of the average cloud cover each year across our study area.

*Table S1:* Original land cover classifications and subsequent reclassifications.

| LC code | Class | Reclassification |
| --- | --- | --- |
| 2001 | Primary Dry Land Forest | MoF_Pri_2nd_dry_Forest |
| 2002 | Secondary Dry Land Forest | MoF_Pri_2nd_dry_Forest |
| 2004 | Primary Mangrove Forest | MoF_Swamp_wet_forest |
| 2005 | Primary Swamp Forest | MoF_Swamp_wet_forest |
| 20041 | Secondary Mangrove Forest | MoF_Swamp_wet_forest |
| 20051 | Secondary Swamp Forest | MoF_Swamp_wet_forest |
| 2014 | Bare Land | MoF_Scrubland |
| 20095 | Transition | MoF_Scrubland |
| 20122 | Transition | MoF_Scrubland |
| 2007 | Scrubland | MoF_Scrubland |
| 3000 | Savannah | MoF_Scrubland |
| 20091 | Dry Rice Land | MoF_Rice_land |
| 20092 | Dry Rice Land Mixed w/Scrub | MoF_Rice_land |
| 20093 | Rice Land | MoF_Rice_land |
| 2010 | Plantation | MoF_Plantation |
| 20071 | Swamp Shrubland | MoF_swamp_scubland |
| 2012 | Housing | MoF_Settlements |
| 20121 | Airport | MoF_Settlements |
| 20141 | Mining | MoF_Settlements |
| 2500 | Cloud | MoF_water_cloud |
| 5001 | Bodies of Water | MoF_water_cloud |
| 20094 | Fish Pond | MoF_water_cloud |
| 5003 | Ocean / River | MoF_water_cloud |
| 50011 | Swamp | MoF_water_cloud |

*Table S2*. Predictor variable labels, descriptions, and group classes as shown in Figure 8.

| Variable label | Variable description | Variable group class |
| --- | --- | --- |
| dist2sett | Distance to settlements | Anthropogenic |
| dist2canal | Distance to canals / rivers | Anthropogenic |
| dist2road | Distance to roads | Anthropogenic |
| Forest_clear_index | Forest clearance index | Land cover |
| Swamp_shrubland | Land cover designation swamp shrubland | Land cover |
| Rice_land | Land cover designation rice agriculture | Land cover |
| Swamp_wet_forest | Land cover designation wet swamp forest | Land cover |
| Scrubland | Land cover designation scrublands | Land cover |
| Water_cloud | Land cover designation water or cloud cover | Land cover |
| Settlements | Land cover designation settlements | Land cover |
| Plantation | Land cover designation plantations | Land cover |
| Peat_d | Peat depth | Environmental |
| ONI | Mean ONI for July - September | Environmental |
| SRTM | Elevation | Environmental |
| ET:PET_[fs/pre_fs] | ET:PET ratio for either fire season (fs) or pre fire season (pre_fs) | Vegetation |
| EVI_[fs/pre_fs] | EVI for either fire season (fs) or pre fire season (pre_fs) | Vegetation |
| NDVI_[fs/pre_fs] | NDVI for either fire season (fs) or pre fire season (pre_fs) | Vegetation |
| SPEI_3mnth_[MONTH] | 3 month Standardised Precipitation Evapotranspiration Index looking back from [MONTH] | Vegetation |
| norm_[variable] | The normalised version of [variable] against a reference area (see methods for details) | Vegetation |
| [variable]_difference | Taken as the difference between August and July measurements of the variable (for M_pre only) | Vegetation |


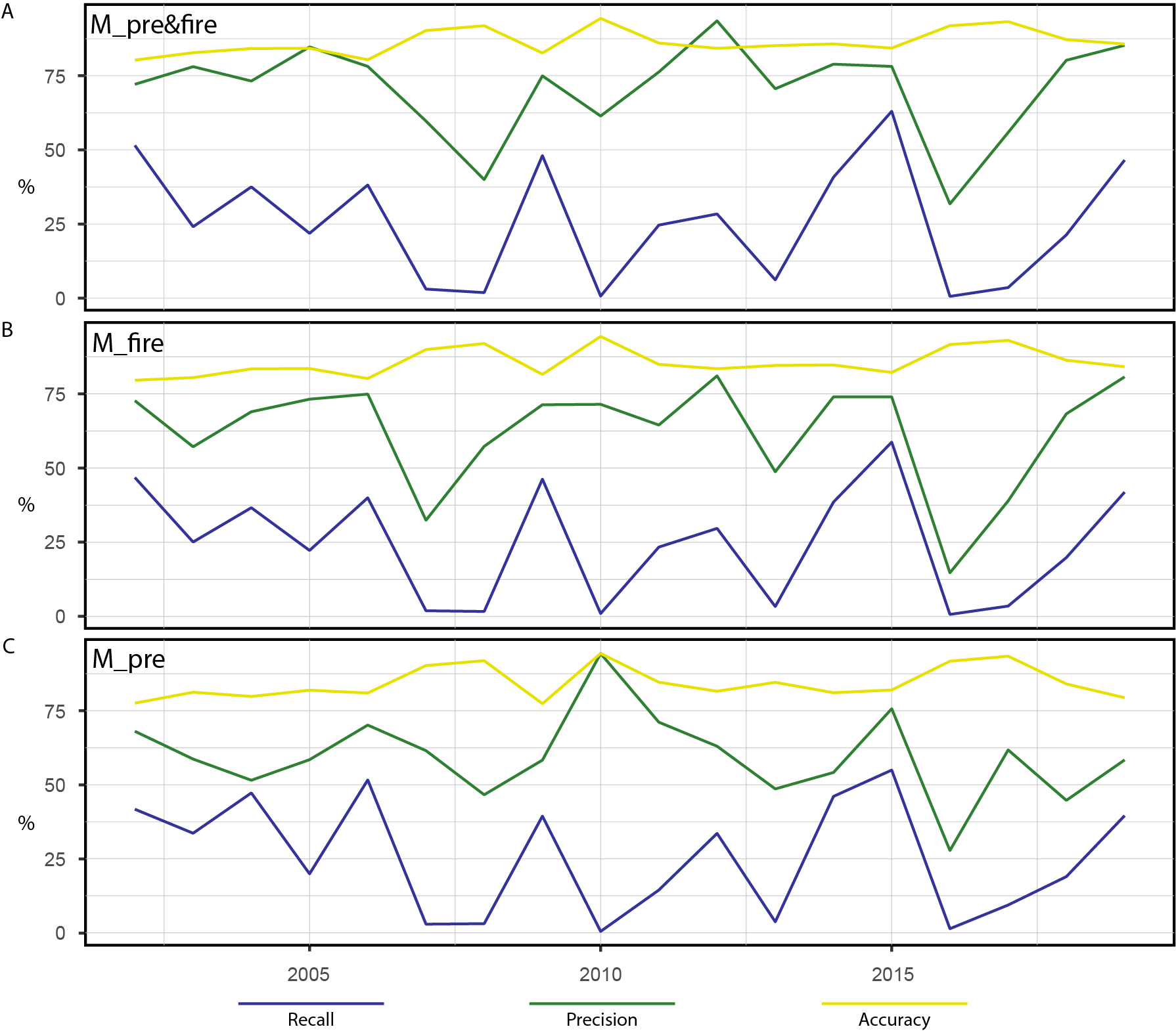


*Figure S1*. Recall (blue line), Precision (green line), and Accuracy (yellow line) for years 2002-2019 for A) model M_pre&fire, B) model fire, and C) model M_pre.

*Figure S2*. (A) Average number
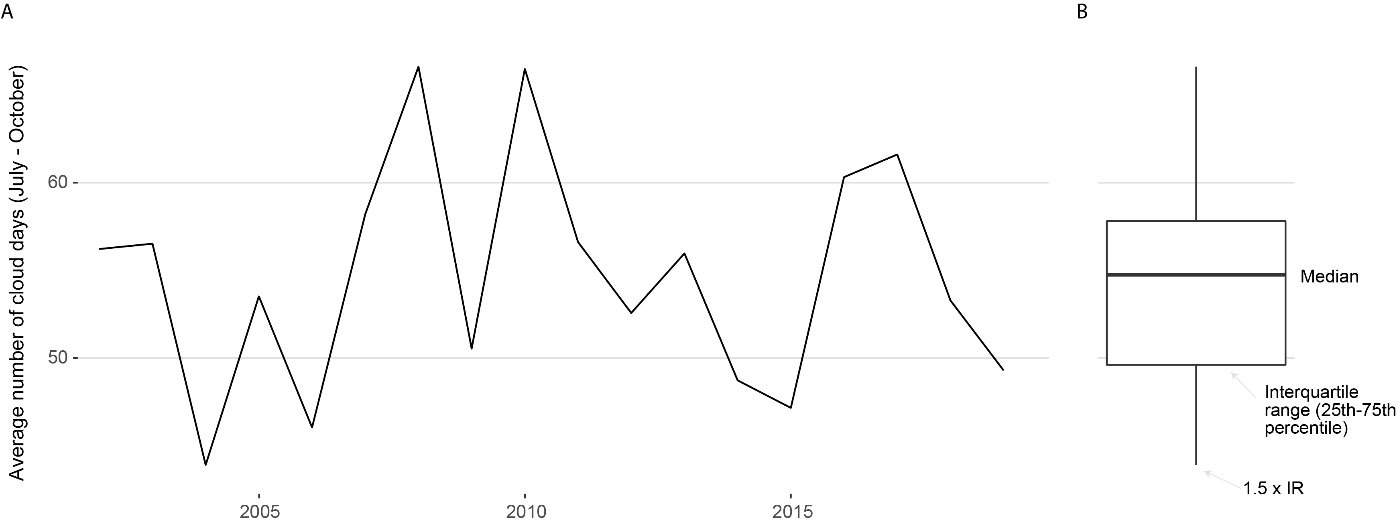
 of cloud days across the study area between July and October for each year (2002 – 2019) as defined by the 1km scale MODIS terra product (MOD09GA version 6). (B) Boxplot showing the median, interquartile range, and whiskers (1.5 x IR) of the data shown in (A).
